# The Role and Interactive Mechanism of Endoplasmic Reticulum Stress and Ferroptosis in Musculoskeletal Disorders

**DOI:** 10.3390/biom14111369

**Published:** 2024-10-28

**Authors:** Zhou Guo, Ruimin Chi, Yawen Peng, Kai Sun, Haigang Liu, Fengjing Guo, Jiachao Guo

**Affiliations:** 1 Department of Orthopaedics, Tongji Hospital, Tongji Medical College, Huazhong University of Science and Technology, Wuhan 430030, China; doc_gzhou@163.com (Z.G.); 18702767740@163.com (K.S.); 15629010092@163.com (H.L.); 2 Department of Rehabilitation, Tongji Hospital, Tongji Medical College, Huazhong University of Science and Technology, Wuhan 430030, China; crm1020@163.com; 3 Department of Obstetrics and Gynecology, Tongji Hospital, Tongji Medical College, Huazhong University of Science and Technology, Wuhan 430030, China; pywen2020@163.com; 4 State Key Laboratory of Reproductive Medicine, The Center for Clinical Reproductive Medicine, The First Affiliated Hospital of Nanjing Medical University, Nanjing 210029, China; 5 Department of Pediatric Surgery, Tongji Hospital, Tongji Medical College, Huazhong University of Science and Technology, Wuhan 430030, China

**Keywords:** endoplasmic reticulum stress, ferroptosis, MSDs, iron homeostasis, lipid metabolism

## Abstract

Endoplasmic reticulum (ER) stress is a cellular phenomenon that arises in response to the accumulation of misfolded proteins within the ER. This process triggers the activation of a signalling pathway known as the unfolded protein response (UPR), which aims to restore ER homeostasis by reducing protein synthesis, increasing protein degradation, and promoting proper protein folding. However, excessive ER stress can perturb regular cellular function and contribute to the development of diverse pathological conditions. As is well known, ferroptosis is a kind of programmed cell death characterized by the accumulation of lipid peroxides and iron-dependent reactive oxygen species (ROS), resulting in oxidative harm to cellular structures. In recent years, there has been increasing evidence indicating that ferroptosis occurs in musculoskeletal disorders (MSDs), with emerging recognition of the complex relationship between ER stress and ferroptosis. This review presents a summary of ER stress and the ferroptosis pathway. Most importantly, it delves into the significance of ER stress in the ferroptosis process within diverse skeletal or muscle cell types. Furthermore, we highlight the potential benefits of targeting the correlation between ER stress and ferroptosis in treating degenerative MSDs.

## 1. Introduction

Musculoskeletal disorders (MSDs) encompass a wide range of conditions that affect the muscles, bones, joints, and associated tissues. These disorders lead to pain, impaired mobility, and functional limitations. In MSDs, inflammation often becomes chronic and contributes to the disease’s progression. In recent years, emerging evidence suggests that endoplasmic reticulum (ER) stress and ferroptosis are also involved in MSDs and contribute to inflammation-mediated tissue damage [1].

The ER is a complex organelle of planate membrane-bound sacs and tubules that plays a critical role in calcium signalling, protein folding, and lipid biosynthesis. The ER is divided into two regions—the smooth ER, which lacks ribosomes and is responsible for lipid metabolism and detoxification, and the rough ER, which is studded with ribosomes and is involved in protein synthesis and maturation [2]. ER stress is a physiological state that arises from an imbalance between the capacity of the ER and the protein folding load; it can be induced by diverse cellular insults, including viral infection, redox imbalance, nutrient deprivation, or genetic mutations [3]. These events induce the massive production of unfolded or misfolded proteins in the ER lumen, triggering the UPR to restore ER homeostasis. However, prolonged or severe ER stress can trigger cell death [4]. As a complex signalling pathway that can mitigate ER stress, the UPR is regulated by three key transmembrane proteins—Inositol-requiring enzyme type 1α (IRE1α), pancreatic ER kinase (PKR)-like ER kinase (PERK), and the activation of transcription factor 6 (ATF6), located in the ER transmembrane [5].

Ferroptosis is a kind of regulated cell death that occurs when lipid peroxides accumulate and damage cell membranes, causing cellular dysfunction [6,7]. This process is triggered by the iron-dependent production of reactive oxygen species (ROS) and ultimately leads to the breakdown of cellular homeostasis. Ferroptosis has been linked to a variety of physical processes and diseases, including inflammation, neurodegeneration, and cancer [6,8,9]. Moreover, recent studies have shown that ferroptosis also contributes to the pathogenesis of osteoarthritis (OA), which is a common joint disease affecting millions of people globally [10]. Chondrocytes, being the principal cellular constituents of articular cartilage, exhibit a heightened susceptibility to ferroptosis owing to their low antioxidant capacity and high metabolic activity [9,11].

Interestingly, recent investigations have revealed a potential connection among ER stress and ferroptosis [12]. When ER stress persists, UPR signalling can promote ferroptosis by upregulating the genes involved in iron uptake, storage, and utilization, as well as inhibiting the genes that regulate antioxidant defence and fatty acid metabolism [13,14,15]. In particular, the ATF4/CHOP pathway, a downstream pathway of UPR, has been associated with the induction of ferroptosis by transcriptionally activating crucial mediators like ACBD6 and SLC7A11 [16,17]. These genes can regulate the availability and transport of intracellular glutathione (GSH), which is an important antioxidant molecule that protects against lipid peroxidation [18,19]. Overall, the relationship between ferroptosis and ER stress highlights the intricate interplay between cellular stress responses and the regulation of oxidative and lipid metabolism.

This paper explores the interplay between ER stress and ferroptosis, as well as the roles of these processes in MSDs. The aim is to provide new insights that may contribute to the prevention and treatment of degenerative joint disorders.

## 2. ER Stress

ER stress is characterized by the condition in which the ER is overwhelmed with unfolded or misfolded proteins, resulting in the disturbance of regular cellular processes [2]. In response to ER stress, cells activate the UPR pathway, which can restore normal ER function by reducing the amount of newly synthesized proteins, increasing the production of chaperones that assist with protein folding, and promoting the degradation of misfolded proteins [20].

**Figure 1:**
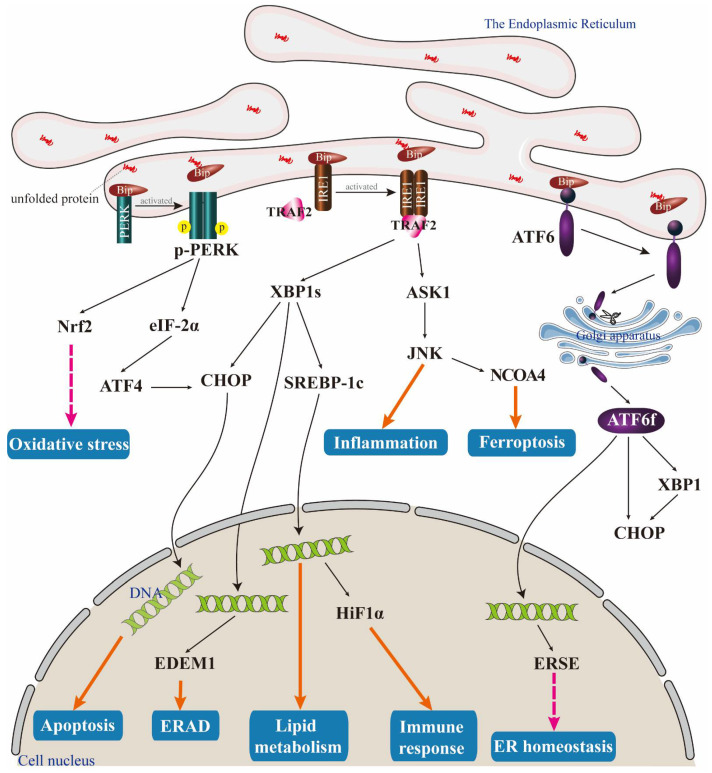
Unfolded protein response induced by ER stress. When cells are stimulated to induce ER stress, the amount of non-folded or misfolded proteins increase, and competitive binding Bip leads to the dissociation and activation of membrane proteins PERK, IRE1α, and ATF6. PERK not only activates the Nrf2 pathway to regulate cellular oxidative stress after autophosphorylation, but also activates the ATF4-CHOP pathway to promote apoptosis through the phosphorylation of eIF-2α. The oligomerization of IRE1α activates RNase activity and promotes the expression of XBP1s. XBP1 can be used as a transcription factor to regulate the expression of CHOP and SREBP-1c, thereby regulating lipid metabolism, endoplasmic reticulum-related degradation, and apoptosis. In addition, activated IRE1α can activate the TRAF2-ASK1-JNK pathway, which plays a role in regulating inflammatory responses and intracellular iron autophagy. After dissociation from Bip, ATF6 translocates to the Golgi apparatus and is cleaved by proteolytic enzymes to produce active ATF6f. ATF6f can enhance CHOP expression, with or without the aid of XBP1, in addition to promoting the transcription of the genes associated with ER-related degradation (ERAD) and lipid metabolism.

The UPR is composed of three branches, each of which is mediated by a specific ER transmembrane protein—IRE1α, PERK, and ATF6 (Figure 1). The IRE1α pathway activates its transcriptional activity by splicing X-box binding protein 1 (XBP1) mRNA, which, in turn, regulates the expression of the genes involved in protein folding and ER-associated degradation (ERAD) [21]. In addition, IRE1α can activate the c-JunN-terminal kinase (JNK) pathway by binding and activating tumour necrosis factor receptor-related factor 2 (TRAF2) [22,23], which can stimulate inflammatory responses and regulate intracellular iron autophagy [24,25]. 

The PERK pathway phosphorylates eukaryotic translation initiation factor 2 α (eIF2 α), which can cause the selective translation of specific mRNAs or global translation inhibition, such as that of ATF4, an important protein that regulates oxidative stress and amino acid metabolism [26]. Additionally, the PERK pathway has the ability to regulate the activity of nuclear factor erythroid 2-related factor 2 (Nrf2) and the mammalian target of the rapamycin complex 1 (mTORC1), thereby controlling cellular metabolism and growth [27].

ATF6 is activated through the liberation of its cytoplasmic domain, facilitating its migration to the nucleus and instigating gene expression programmes that foster UPR. The process involves two distinct steps—trafficking from the ER to the Golgi and proteolytic cleavage within the Golgi [28,29]. In addition to its transcriptional activity, ATF6 possesses the capability to engage in interactions with other components of UPR, including IRE1α and PERK, thereby facilitating the coordination of the UPR.

## 3. Ferroptosis 

**Figure 2:**
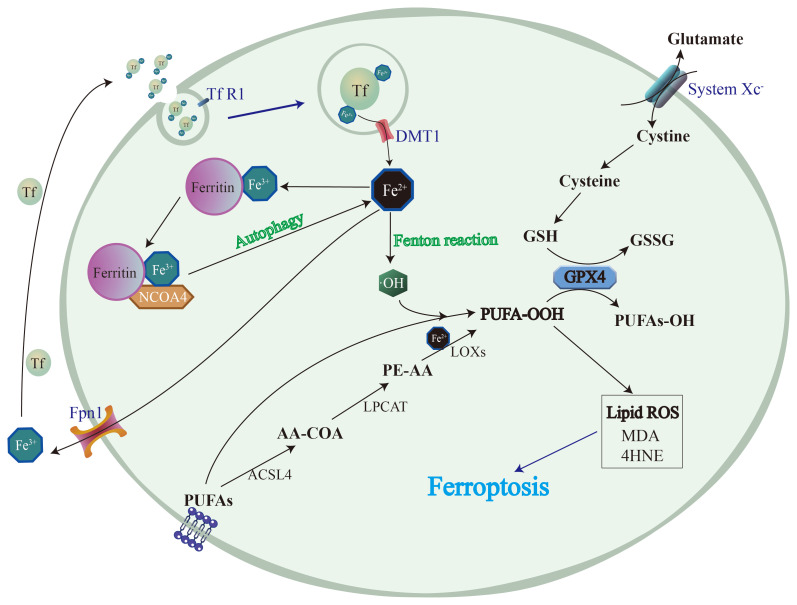
The regulation of ferroptosis. The regulatory signals of ferroptosis can be categorized into three main types—iron metabolism, the GSH/Gpx4 pathway, and lipid peroxidation. An imbalance in iron metabolism can lead to the accumulation of free iron within chondrocytes, which further triggers the generation of lipid free radicals through the Fenton reaction, resulting in lipid peroxidation. Cysteine is an essential component for the synthesis of GSH and GSH peroxidase 4 (Gpx4), and plays a crucial role in reducing lipid peroxidation. Hence, the inhibition of Gpx4 production can contribute to ferroptosis. Furthermore, phospholipid metabolism is also closely associated with ferroptosis. The enzymes involved in lipid metabolism, such as ACSL4 and LOX15, participate in the production of peroxidized unsaturated fats from membranous phospholipids. These peroxidized lipids cause cellular damage and promote ferroptosis.

Ferroptosis is a type of regulated cell death that has recently been discovered. It is initiated by an imbalance between oxidants and antioxidants within cells, leading to an increase in ROS production. This triggers a cascade of events, resulting in the formation of unstable lipid peroxides that can quickly spread throughout the cell and cause significant damage (Figure 2). 

Despite being a relatively new field, researchers have made significant progress in studying ferroptosis in recent years. Several key genes and pathways that regulate ferroptosis have been identified, such as the GSH system, which plays a critical role in regulating redox balance [30]. Lipid peroxidation, which is a key feature of ferroptosis, is initiated by the depletion of GSH, a major antioxidant molecule that is synthesized from cysteine obtained through the diet or uptake via the system xc-. The inhibition of this antiporter leads to cysteine deprivation, which results in GSH depletion and an increased susceptibility to oxidative stress [31]. Another one of the pivotal components involved in ferroptosis is iron. While it is an essential element for many cellular processes, excess iron accumulation can result in toxicity [32]. The accumulation of iron within cells may occur due to a variety of factors, such as altered iron metabolism, impaired autophagy, and defective iron export pathways [9]. Additionally, during oxidative stress, iron may be released from ferritin and other intracellular iron-binding proteins. In the context of ferroptosis, iron plays a critical role in the production of lipid peroxides [33]. Another crucial pathway involved in ferroptosis is the phospholipid metabolism pathway, which regulates cell membrane composition and contributes to lipid peroxide formation. The accumulation of lipid peroxides is another factor in ferroptosis. Lipid peroxidation is a multifaceted process that involves the oxidation of polyunsaturated fatty acids (PUFAs) in the cell membrane [34]. This process results in the formation of various lipid peroxides, such as malondialdehyde (MDA) and 4-hydroxynonenal (4-HNE) [35]. These peroxides can react with other molecules within the cell, including proteins and DNA, resulting in additional damage. In conclusion, ferroptosis is a recently discovered form of regulated cell death characterized by the accumulation of iron-dependent lipid peroxides. It is triggered by a combination of redox imbalance, iron accumulation, and lipid peroxidation. 

## 4. Crosstalk Between ER Stress and Ferroptosis 

**Figure 3:**
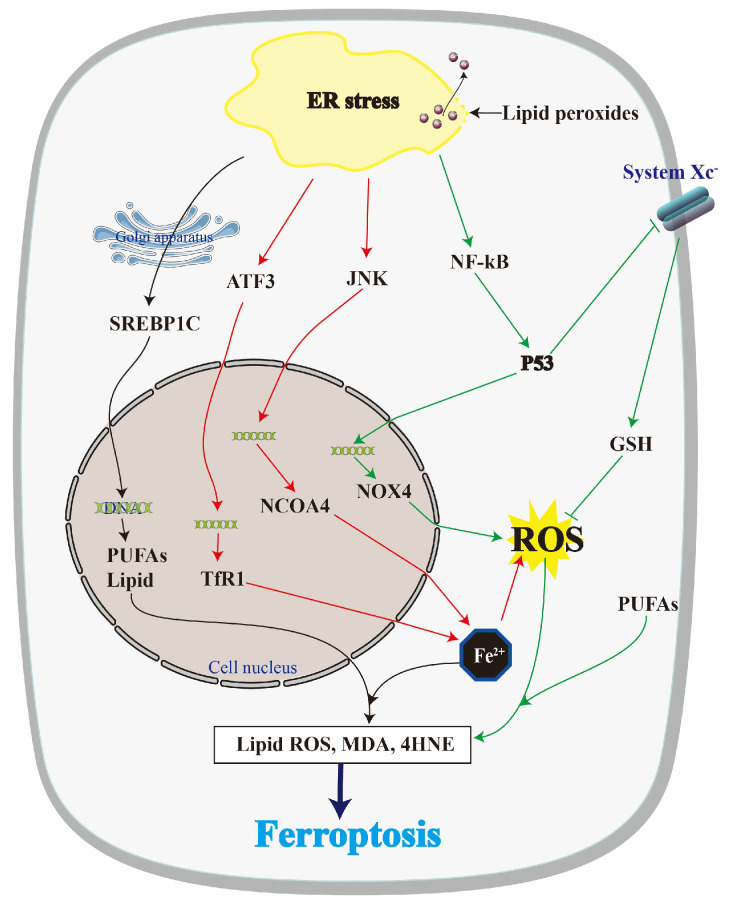
The possible relationship between ER stress and ferroptosis. When ER stress occurs, SREBP-1c on the endoplasmic reticulum plays an active role in regulating lipid metabolism and promoting phospholipid peroxidation through the Golgi apparatus. In addition, the expression of ATF3 and JNK increased after ER stress, as well as increasing the intracellular activity through TfR1 and NCOA4, respectively, affecting the accumulation of ROS and lipid peroxides. The activation of PERK and IRE1α also enhances the expression of p53 through NF-κB, thus destroying the redox homeostasis of cells. On the other hand, lipid peroxides are able to directly destroy the endoplasmic reticulum or damage protein folding and stimulate ER stress.

**Figure 4:**
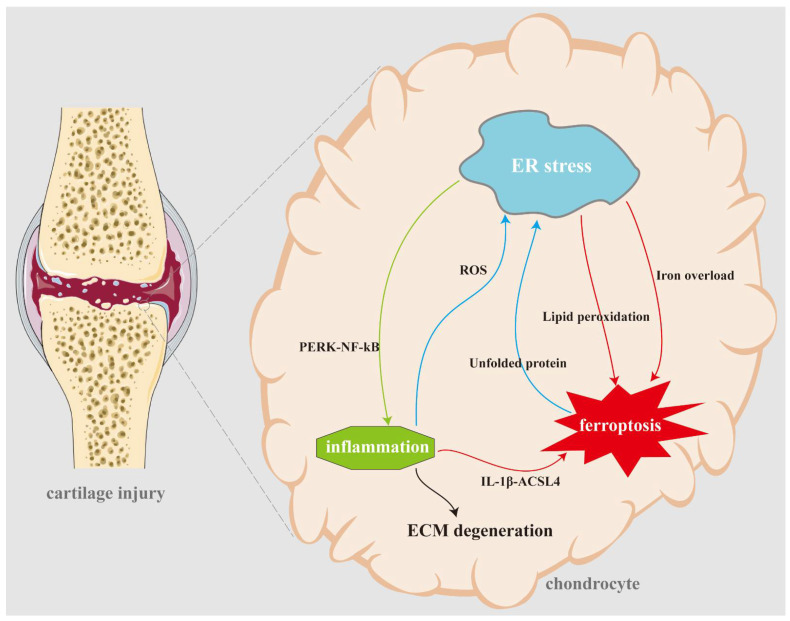
The mechanism of ER stress and ferroptosis in osteoarthritis. When articular cartilage suffers from injury or degradation, it leads to the altered metabolic activity and morphology of chondrocytes. In this pathological scenario, a complex interplay occurs between inflammation, endoplasmic reticulum (ER) stress, and cell ferroptosis, ultimately resulting in chondrocyte death and the degradation of the cartilage matrix. Within the chronic injury environment, chondrocytes experience intracellular inflammation, which triggers the accumulation of reactive oxygen species (ROS) and activates ER stress. Consequently, ER stress stimulates lipid peroxidation and disrupts iron metabolic homeostasis, thereby initiating ferroptosis. Additionally, the PERK-NF-κB pathway is activated, further intensifying intracellular inflammation. Inflammatory responses also contribute to ferroptosis by augmenting the expression of ACSL4. Subsequently, ferroptosis enhances ER stress by upregulating protein unfolding processes.

Recent studies have elucidated that an overabundance of oxidative stress and lipid peroxidation caused by ageing or injury can induce ferroptosis, thereby precipitating the damage of the cartilage and muscle, and playing an irreplaceable role in the development of MSDs [36]. Interestingly, ER stress can induce ferroptosis by dysregulating lipid metabolism, redox balance, and iron homeostasis [37,38]. Conversely, ferroptosis can cause ER stress through various mechanisms, including GSH depletion, ROS generation, and lipid peroxide accumulation [13,39]. The interplay between these processes is intricate and multifaceted, potentially playing a role in the death of cells and the onset and progression of MSDs (Figure 3 and Figure 4).

### 4.1. Regulation of Lipid Metabolism

One of the mechanisms by which ER stress triggers ferroptosis is through the dysregulation of lipid metabolism. Specifically, ER stress can activate the transcription factor SREBP-1c, which controls the genes involved in lipogenesis and increases intracellular lipid levels [40]. SREBP-1c regulates lipogenic genes such as fatty acid synthase (FASN) and acetyl-CoA carboxylase (ACC). Normally, insulin signalling and nutrient availability regulate SREBP-1c, but ER stress can activate it via the UPR pathway. Once activated, SREBP-1c upregulates the expression of lipogenic genes and increases intracellular lipid levels, which can make cells more susceptible to ferroptosis by providing substrates for lipid peroxidation [41]. Lipid peroxidation occurs when ROS or active iron ions attack the double bonds in unsaturated fatty acids, resulting in the formation of reactive aldehydes such as MDA and 4-HNE, as well as lipid hydroperoxides. These reactive species can damage cellular membranes, proteins, and DNA, thereby triggering ferroptosis [42]. Moreover, ER stress can decrease the expression of fatty acid desaturase 1 (FADS1), an enzyme that converts saturated fatty acids to unsaturated fatty acids [43]. This impairs the activity of GPX4, a key enzyme that detoxifies lipid peroxides, ultimately promoting ferroptosis [44].

On the other hand, lipid peroxides can directly damage the ER membrane, which can release calcium ions from the ER lumen into the cytoplasm and then overwhelm the protein folding capacity of the ER [45]. One such mechanism involves the direct modification of ER-resident proteins by lipid peroxidation products. For instance, 4-HNE, which is a major lipid peroxidation product, can modify proteins by forming covalent adducts with cysteine, histidine, and lysine residues. This modification can change protein conformation, activity, and stability, leading to the accumulation of misfolded proteins and the activation of the UPR [46]. Another mechanism by which lipid peroxides can induce ER stress involves the disruption of lipid metabolism. Lipid peroxides can modify metabolism, leading to changes in their physical properties and disrupting membrane structure and function. This disruption can cause misfolded protein accumulation and activate the UPR, resulting in ER stress [47]. Moreover, lipid peroxides can induce ER stress by generating ROS and reactive aldehydes. ROS are highly reactive molecules that can cause oxidative damage to various cellular components, including proteins and lipids, further contributing to ER stress [48]. In summary, lipid peroxidation can induce ER stress through several mechanisms involving the direct modification of ER-resident proteins, the disruption of lipid metabolism, and the generation of ROS and reactive aldehydes. 

### 4.2. Regulation of Redox Balance

One mechanism by which ER stress induces ferroptosis is through the activation of the transcription factor p53. In response to ER stress, p53 promotes the expression of pro-oxidant enzymes such as NADPH oxidase 4 (NOX4), which generates ROS. Interestingly, some intracellular lipid peroxides and toxic lipid hydroperoxides are generated by the reaction of ROS with PUFAs in cell membranes [49]. In recent years, some findings show that the upregulation of p53 leads to a significant decrease in the mRNA and protein expression level of SLC7A11, a component of system xc-, which eventually causes a decrease in GSH synthesis and GSH-dependent GPX4 activity. In summary, ER stress induces ferroptosis by promoting oxidative stress through the p53-mediated activation of NOX4 and the depletion of GSH via system xc- inhibition [50,51].

Ferroptosis has been demonstrated to reduce the levels of GSH, a crucial antioxidant that protects cells from oxidative stress [52]. The reduction in GSH results in an increase in ROS, which can damage lipids, proteins, and DNA. This damage can subsequently lead to the accumulation of unfolded or misfolded proteins within the ER, thereby activating the UPR and ultimately culminating in cell death [53]. Ferroptosis can cause ER stress through another mechanism that involves the generation of ROS, which directly oxidizes the proteins that are present in the ER lumen, causing their misfolding and aggregation [12]. Moreover, ROS can trigger the activation of the UPR by inducing the expression of the transcription factor CHOP [54].

### 4.3. Regulation of Iron Metabolism

Iron is a critical cofactor for lipid peroxidation and is required for the initiation and propagation of ferroptosis [55]. One mechanism by which ER stress can induce ferroptosis is through the upregulation of transferrin receptor 1 (TfR1), a protein involved in iron uptake. While TfR1 is typically expressed at minimal levels in most tissues, its expression can be increased in response to iron deficiency or hypoxia. Research has shown that the ER stress-induced activation of the PERK-eIF2α-ATF4 signalling pathway increases the expression of the activation of transcription factor 3 (ATF3), which, in turn, upregulates TfR1 expression. This process leads to iron accumulation mediated by TfR1 and subsequent ferroptosis in hepatocytes [56]. Conversely, ER stress can downregulate the expression of ferritin, a protein that sequesters iron and prevents its participation in harmful reactions. Ferritin is composed of heavy and light subunits and stores iron in a nontoxic form. Under conditions of ER stress, the expression of ferritin can be suppressed, leading to increased levels of labile iron and the initiation of ferroptosis [37,57].

In addition, previous studies have shown that the JNK-JUN-NCOA4 axis contributes to iron-mediated chondrocyte death and exacerbates OA through ferritinophagy [58]. Importantly, IRE1α can stimulate JNK activation via TRAF2, further promoting iron-mediated cell death [59]. This finding highlights the specific connection between ER stress and iron-mediated ferroptosis. Additionally, iron plays a crucial role in initiating and propagating lipid peroxidation. In the presence of iron, lipid peroxides can undergo the Fenton reaction, generating highly reactive hydroxyl radicals that can cause extensive damage to cellular components [60]. Under conditions of ER stress, dysregulated iron homeostasis can promote iron-dependent lipid peroxidation and subsequent ferroptosis [61]. These findings suggest that the ER stress-induced dysregulation of iron homeostasis can promote ferroptotic cell death through the induction of lipid peroxidation. Furthermore, recent studies have shown that PERK activates p53 by phosphorylating histone H2AX (γ H2AX) serine 139 and accumulating it in the nucleolus [62], which would upregulate the expression of TfR1 and promote ferritin autophagy, thus promoting ferroptosis. 

There are also recent articles investigating the effects of disturbed iron metabolism on the cellular ER stress response and ferroautophagy [38]. Some of these results suggest that acute iron overload leads to increased mitochondrial ROS (mtROS) production, lipid peroxidation, impaired autophagic flux, and ferroptosis. Reducing ER stress with 4-phenylbutyric acid (4-PBA) improved cellular autophagic flux and ferroptosis. In conclusion, iron overload causes different forms of impaired ferroautophagy and ferroptosis, partly through the mechanism of ER stress.

## 5. The Bridge Between MSDs and Ferroptosis: ER Stress

Most of the skeletal muscle diseases are age-related degenerative changes; thus, oxidative stress and metabolic disorders are important factors in their onset and progression. Although ferroptosis has been explored in an increasing number of diseases in recent years, the precise understanding of the involvement of ferroptosis in different cell types and its response mechanism to ER stress in MSDs remains limited. This section aims to elucidate the specific role and interactive mechanism of ER stress and ferroptosis in orthopedic diseases, drawing upon previous research findings.

### 5.1. Osteoarthritis

Research has shown that ER stress is increased in chondrocytes during OA. The UPR responds to ER stress in chondrocytes and is activated, which is associated with the increased expression of the genes involved in inflammation and cartilage degradation [63]. Lipid peroxidation is another factor that promotes the pathogenesis of OA. In ageing chondrocytes or in a chronic inflammatory environment, lipids, such as fatty acids and cholesterol, within cells are susceptible to oxidative damage caused by ROS. This process produces lipid peroxides, which further damage cell membranes and promote inflammation. The accumulation of oxidized lipids can induce iron-mediated chondrocyte death and activate inflammatory pathways, leading to further cartilage degradation [64]. Overall, ER stress and lipid peroxidation play important roles in the development and progression of OA.

Similar to other cells, ER stress in chondrocytes is complex to cross with ferroptosis signals. Some studies reported, through bioinformatics analysis combined with cellular experimental validation, that the ER stress response factors CXCL2, ATF3, and the iron transport protein TFRC were upregulated in IL-1β-stimulated chondrocytes, whereas JUN and c-JUN were downregulated, which initially revealed a potential link between ER stress and ferroptosis in chondrocytes during the pathological development of OA; additionally, iron metabolism and lipid peroxidation may play an important role in it [65,66]. Gong Z et al. found that Cardamonin (CAD) alleviated chondrocyte mitochondrial and ER function through the P53 signalling pathway, regulated ferroptosis, and improved the degradation of OA cartilage [67]. P53 can not only stimulate inflammation, but also regulate GPX4 expression through SLC7A11. This study further confirmed the important role of the p53 pathway in the relationship between oxidative stress and ferroptosis in chondrocytes. In addition, studies by He and others have also shown that Biochanin A (BCA) inhibited oxidative stress and the ferroptosis of chondrocytes by alleviating iron overload. Specifically, ferric ammonium citrate (FAC) treatment for one day resulted in an increase in intrachondrocyte iron, ROS, and lipid ROS content, as well as an accumulation of ER damage markers, but recovered after BCA treatment. Meanwhile, the results indicated that BCA at a concentration of 24 μM significantly activated the Nrf2/system xc-/GPX4 signalling pathway and inhibited TfR1 expression, which directly reduced intrachondrocyte iron concentration, decreased oxygen free radical accumulation, and prevented lipid peroxidation [16]. Thus, ER stress and ferroptosis often occur in tandem and play an important role in the development and progression of OA. Further studies are needed to fully understand the mechanisms behind these processes and to explore potential therapeutic targets for the treatment of OA.

### 5.2. Intervertebral Disc Degeneration

The anatomical structures of the intervertebral disc (IVD) from the inside to the outside include, respectively, the internal nucleus pulposus (NP), the external annulus fibrosus (AF), and the upper and lower cartilage endplates (CEPs). A major pathological progression of intervertebral disc degeneration (IVDD) is the destabilization of the NP and cartilage endplate degeneration, which is a pathologic change that dramatically alters the biomechanics and nutrient supply status of the disc. Similar to osteoarthritis, degenerated intervertebral discs are often accompanied by iron overdeposition due to age-related pathological changes or tissue damage caused by multiple stressors, and oxidative stress and ferroptosis caused by iron overdeposition promote NP cell reduction, endplate chondrocyte senescence, and cartilage degradation to some extent [68]. Extracellular matrix (ECM) homeostasis is very important for IVD stabilization, and the IVD microenvironment is quite intricate, in which the ER in various types of cells is highly susceptible to external stimuli, inducing the occurrence of ER stress-mediated ferroptosis and ECM denaturation, and ultimately initiating and facilitating the progression of IVDD [69].

In a recent epidemiologic study, iron overload was found to be an independent risk factor for IVDD in humans [70]. Using iron overload mouse models, Wang Wenchao et al. found that iron promoted IVDD and endplate cartilage degeneration in a dose-dependent manner. Whereas, in vitro, 4-PBA and a ferroptosis inhibitor (Fer-1) effectively inhibited iron overload-induced endplate chondrocyte degeneration; these findings further confirmed the promoting role of ER stress and ferroptosis in IVDD progression [68]. An interesting recent experiment found that the use of materials engineering-assisted polydopamine nanoparticles inhibits ER stress in NP cells (NPCs) and chelates intracellular ferrous ions, regulating ferritin expression, thereby decreasing MDA and lipid peroxidation production and inhibiting the ferroptosis of NPCs [71]. This study preliminarily suggested the role of ER stress in regulating iron metabolism and thereby regulating ferroptosis in NP cells. In addition, it has been shown that tert-butyl hydroperoxide (TBHP) induces ferroptosis in myeloid and annular fibroblasts (AFCs) via ER stress, whereas the knockdown of NCOA4 inhibited ferroptosis in AFCs and NPCs via ferritin-selective autophagy. This further confirmed the important role of NCOA4-mediated ferritinophagy in the connection between ER stress and ferroptosis.

### 5.3. Osteoporosis

Osteoporosis is characterized by increased bone fragility due to decreased bone density and mechanical strength, which is caused by decreased bone formation relative to bone resorption. Osteoblasts are the central cells of the bone formation process, and abnormal iron metabolism not only inhibits the osteogenic differentiation of bone marrow mesenchymal stem cells, but also negatively affects the activity of mature osteoblasts and extracellular matrix mineralization [72]. In addition, ER stress mediates the IRE1α signalling pathway and the NF-κB signalling pathway to promote osteoclastogenesis, which initiates osteoporosis caused by the excessive differentiation of osteoclasts [73].

In recent years, more and more studies have shown that iron overload is an independent risk factor for osteoporosis. Iron overload causes mitochondrial damage and ROS elevation and activates ER stress [74]. The NF-κB pathway initiated by PERK phosphorylation not only induces inflammatory factors, but also activates the Smad and MAPK signalling pathways in osteoblasts to inhibit osteogenic differentiation [75]. These findings confirmed that the interaction between ER stress and ferroptosis is closely related to osteogenic differentiation and osteoporosis progression. At the same time, the UPR after ER stress inhibits Wnt signal transduction, downregulates the expression of Nrf2, and promotes the ferroptosis of osteoblasts through the Nrf2-ARE signal pathway [76]. In the study of Tsay et al., it was found for the first time that the level of oxidative stress and bone resorption increased in iron-overloaded mice, while the treatment of antioxidant N-Acetyl-L-cysteine (NAC) reversed the development of bone loss in iron-overloaded mice [77]. Specifically, excessive iron uptake stimulates ER stress and ROS accumulation, further activates the MAPKs or NF-κB pathway, and promotes osteoclast production and bone loss [43]. Combined with previous studies, it is not difficult to find that, on the one hand, ER stress is involved in bone marrow mesenchymal stem cell (BMSC) differentiation and osteoblast ferroptosis induced by iron metabolism disorders. On the other hand, ER stress also directly affects the activity of osteoblasts and osteoclasts, and regulates the progress of osteoporosis by regulating various signal pathways to participate in the balance formation of osteoblasts and osteoclasts [78]. 

### 5.4. Sarcopenia

The main mechanism of sarcopenia (SP) is a degenerative decline in skeletal muscle mass and strength caused by an imbalance in muscle synthesis and degradation, which is common in the elderly or patients with cachexia [79]. The exact cause of SP is still unclear, but may include redox imbalance in skeletal muscle, immune factors, hormone disorders, malnutrition, and reduced physical activity [80]. Sarcoplasmic reticulum is an important organelle of skeletal muscle cells, and it is a special ER. During oxidative stress, the mismatch of protein synthesis, degradation in the sarcoplasmic reticulum, and the disturbance of calcium transport are the main factors leading to SP [81]. A recent study by Shengmin Lee et al. demonstrated that skeletal muscle cells activated the PERK-eIF2α pathway via FABP3-dependent lipid peroxidation to cause ER stress and reduced protein synthesis to inhibit muscle recovery after immobilization [82]. Finally, Huang Yan et al. found excessive iron accumulation in both patients and animal models of sarcopenia. Using 40-week-old senescence-accelerated mouse prone 8 (SAMP8) mice as models of sarcopenia, they found that iron overload in muscle tissue induced ER stress in muscle cells and upregulated the expression of P53, which inhibited the protein level of SLC7A11, leading to increased intracellular lipid peroxidation, causing ferroptosis [83].

## 6. Therapeutic Prospects and Current Limitations

These studies indicate that normal ER function and iron homeostasis are crucial for cell survival. Cellular damage or developmental disorders caused by ER stress and/or iron metabolism disorders are associated with the development of most MSDs. Thus, potential interventional approaches to maintain ER function and iron homeostasis can be broadly categorized into three groups, as follows: the regulation of the p53 pathway, the modulation of redox homeostasis, and the regulation of iron metabolism (Table 1).

### 6.1. Regulation of the p53 Pathway

p53 is a transcription factor that plays a role in the regulation of ER stress-mediated ferroptosis. P53 not only affects the GSH level of cells by regulating SLC7A11 and P21, but also activates the SAT1-ALOX15 axis to promote the lipid peroxidation process [91,92]. Therefore, blocking the signal transduction of the P53 pathway is an effective strategy to break the vicious cycle of ER stress and ferroptosis, as well as providing a new therapeutic approach to prevent ferroptosis and treat MSDs.

The dysregulation of p53 can contribute to the development of MSDs by promoting the ferroptosis of osteocytes and the degradation of cartilage [93,94]. There is evidence that certain drugs may have protective effects on chondrocytes by regulating the activity of p53, a protein that plays a key role in cell growth and death regulation [95]. 

Resveratrol is a natural compound found in grapes, berries, and other plants. It has anti-inflammatory and antioxidant properties, which enable it to inhibit both ER stress and ferroptosis. The latest study has preliminarily confirmed the ability of resveratrol to protect BMSC from osteogenic differentiation by regulating p53, and shows its potential in the treatment of osteoporosis [84]. Furthermore, resveratrol has been shown to inhibit p53 activity in chondrocytes and protect against cartilage damage in animal models of OA [96]. Resveratrol has also been shown to protect against acrolein-induced ferroptosis in MIN6 cells via the ER stress-associated PERK pathway [13]. CAD, another natural compound found in turmeric with anti-inflammatory and antioxidant properties, has been studied for its potential to regulate p53 activity in chondrocytes and protect against cartilage degradation in animal models of OA [67].

However, further research is needed to fully understand the mechanism of these drugs on chondrocyte p53 activity and their potential use in treating MSDs.

### 6.2. Modulation of Redox Homeostasis 

Redox imbalance plays an important role in the occurrence of ferroptosis, making it a potential therapeutic target for treating MSDs. Several drugs have been shown to protect cartilage or skeletal muscle by regulating oxidative stress [97,98]. 

Melatonin, a naturally occurring hormone with antioxidant properties, has been shown to inhibit lipid peroxidation and protect chondrocytes from oxidative stress-induced damage [87]. Recent studies have demonstrated that melatonin can regulate the Nrf2 or yap pathway to protect cardiomyocytes or treat age-related cataracts [99,100]. Taken together, melatonin is a potentially effective drug for redox homeostasis to regulate the interaction between ER stress and ferroptosis in MSDs. Zhao et al. recently found that fucoidan hydrogels can ensure the production of ECM in chondrocytes through ROS scavenging, and realize cartilage regeneration [101]. This finding not only contributes to the treatment of osteoarthritis, but also has significance for the treatment of epiphyseal plate injury in children and degenerative cartilage injury of IVD in the elderly. Vitamin E, a fat-soluble vitamin with antioxidant properties, has also been shown to inhibit lipid peroxidation and protect chondrocytes from oxidative stress-induced damage [89]. Curcumin, a natural compound found in turmeric with antioxidant and anti-inflammatory properties, has been shown to inhibit lipid peroxidation and protect chondrocytes from oxidative stress-induced damage [102].

Further in vivo and clinical research is needed to fully evaluate the safety and efficacy of these drugs and their ability to maintain redox balance.

### 6.3. Regulation of Iron Overload

The regulation of iron metabolism has been shown to inhibit ER stress and ferroptosis. Iron plays a crucial role in the pathogenesis of MSDs, and excessive iron accumulation can induce ER stress or trigger ferroptosis directly via the Fenton reaction [10,103]. Therefore, targeting iron metabolism may provide a therapeutic approach for preventing ER stress and ferroptosis in MSDs.

One approach to regulate iron metabolism is through the use of iron chelators, which can reduce iron levels and prevent the accumulation of ROS that contribute to oxidative stress and ferroptosis. The latest research confirmed that the iron chelator deferoxamine could effectively inhibit iron overload in chondrocytes and reduce ferroptosis in chondrocytes in vivo and in vitro, thereby reducing the progression of OA [11]. Similarly, the use of iron chelating agents or ferroptosis inhibitors has been shown in recent years to have certain therapeutic effects on IVDD and osteoporosis by maintaining iron homeostasis and inhibiting cellular oxidative stress [68,72,104]. Another approach is to target the proteins involved in iron metabolism, such as transferrin, ferritin, and hepcidin, which regulate iron uptake, storage, and release in cells. By modulating the expression or activity of these proteins, it may be possible to prevent the accumulation of excess iron and protect cells from ER stress and ferroptosis [105]. For example, BCA directly reduces intracellular iron concentration, scavenges free radicals, and prevents lipid peroxidation by inhibiting TfR1 and promoting FPN [106]. There are also polydopamine nanoparticles (PDANPs) fabricated using materials engineering means to antagonize ferroptosis in NP cells by scavenging ROS, chelating Fe^2+^ to attenuate iron overload, and further downregulating MDA and lipid peroxide production, thus significantly delaying IVD degeneration. [71].

Overall, the regulation of iron metabolism represents a promising therapeutic strategy for preventing ER stress and ferroptosis in MSDs and other diseases associated with iron-dependent cells.

## 7. Conclusions and Future Perspectives

In conclusion, ER stress plays a critical co-regulatory role in the induction of ferroptosis in MSDs. Understanding the underlying mechanisms of these processes may lead to the development of novel therapeutic strategies for the treatment of MSDs. Future studies should focus on identifying specific target molecules within ER stress pathways that could be used to modulate ferroptosis and prevent cartilage degradation.

## Figures and Tables

**Table 1:** Regulation of ERS and ferroptosis as potential therapeutic strategies for MSDs.

Regulation Pathway	Drugs	Effect	Reference
p53 pathway	Resveratrol	Resveratrol appears to be a potent in vitro anti-inflammatory agent that alleviates osteoporosis by rescuing p53-inhibited osteogenic differentiation.	[84]
	Hesperidin	Hesperidin reversed osteogenic differentiation inhibited by dexamethasone to some extent by inhibiting p53 activation.	[85]
	Cardamonin	CAD ameliorated OA cartilage degradation by regulating ferroptosis via the P53 signalling pathway.	[67]
	Eupatilin	Eupatilin reduced TNF-α-induced cellular senescence by inhibiting p53 expression in NP cells.	[86]
Redox homeostasis	Melatonin	Melatonin protected chondrocytes via mitochondrial redox homeostasis and autophagy.	[87]
	Fucoidan	Fucoidan protects ECM production in NP cells by activating the nuclear factor erythroid 2-related factor 2 (NRF2) antioxidant system.	[88]
	Vitamin E	Vitamin E has a preventive effect on the lipid peroxidation of chondrocytes and the oxidation and degradation of cartilage matrix proteins.	[89]
	Curcumin	Curcumin could inhibit the PERK-eIF2α-CHOP axis of the ER stress response through the activation of SIRT1 and ameliorated osteoarthritis development in vivo.	[90]
Iron metabolism	Biochanin A	BCA directly reduced intracellular iron concentration, scavenged free radicals, and prevented lipid peroxidation by inhibiting TfR1 and promoting FPN.	[16]
	Polydopamine nanoparticles	PDANPs antagonized ferroptosis in NP cells by scavenging ROS, chelating Fe^2+^ to attenuate iron overload, and further downregulating MDA and lipid peroxide production.	[71]
	Deferoxamine	DFO inhibited chondrocyte ferroptosis by chelating iron ions and promotes the activation of the Nrf2 antioxidant system.	[11]
